# The Landscape of Immunotherapy for Retroperitoneal Sarcoma

**DOI:** 10.3390/curroncol30020165

**Published:** 2023-02-09

**Authors:** Alicia A. Gingrich, Elise F. Nassif, Christina L. Roland, Emily Z. Keung

**Affiliations:** 1 Department of Surgical Oncology, MD Anderson Cancer Center, Houston, TX 77030, USA; 2 Sarcoma Medical Oncology, MD Anderson Cancer Center, Houston, TX 77030, USA

**Keywords:** retroperitoneal sarcoma, immunotherapy, sarcoma immune class, tumor microenvironment, tumor infiltrating lymphocyte, tumor mutational burden

## Abstract

Significant multidisciplinary scientific effort has been undertaken to understand the heterogeneous family of neoplasms that comprise soft tissue sarcomas. Within this family of neoplasms, outcomes for retroperitoneal sarcomas (RPS) are currently limited given a lack of effective therapies. In this review, we focus on immunotherapy and its relationship with the common RPS histologic subtypes. Although initial outcomes for RPS patients with immune checkpoint inhibition alone have been somewhat disappointing, subsequent analyses on histologies, the tumor microenvironment, sarcoma immune class, tumor infiltrating lymphocytes and genetic analysis for tumor mutational burden have yielded insight into the interplay between sarcomas and immunotherapy. Such approaches have all provided critical insight into the environment and characterization of these tumors, with targets for potential immunotherapy in future clinical trials. With this insight, molecularly tailored combination treatments for improving response rates and oncologic outcomes for RPS are promising.

## 1. Background

**Figure 1:**
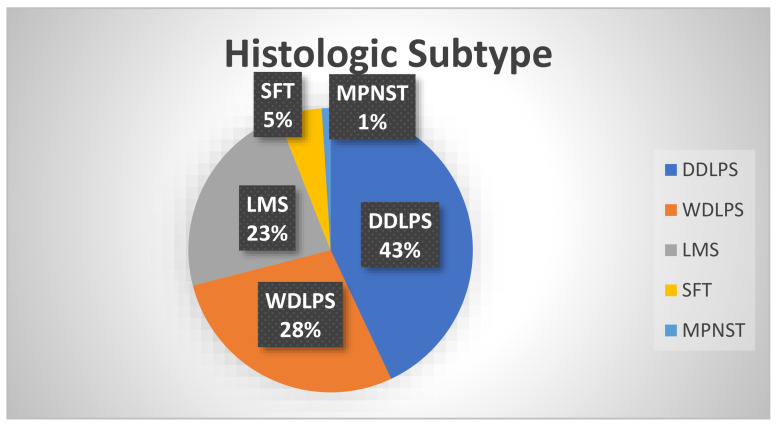
Common retroperitoneal sarcoma histologic subtypes. Pie chart depicting the distribution of sarcoma subtypes found within the retroperitoneum and their frequencies.

Soft tissue sarcomas (STS) are rare mesenchymal neoplasms which can arise from any connective tissue such as muscle, fat, blood vessels, cartilages, and nerves [1]. They comprise an estimated 1% of adult tumors and span over 100 histologic subtypes based on their tissue of origin, histologic appearance, and molecular characteristics [2]. Among STS, 15% arise in the retroperitoneum and these are characterized by distinct clinical behaviors, prognoses, and multimodal management depending on their histology [1,3]. The most common histologic subtypes of retroperitoneal STS (Figure 1) include well-differentiated liposarcoma (WDLPS), de-differentiated liposarcoma (DDLPS), leiomyosarcoma (LMS), solitary fibrous tumor (SFT), and malignant peripheral nerve sheath tumor (MPNST) [3,4].

Macroscopically complete surgical resection remains the standard of care and the only potentially curative treatment for patients with localized resectable STS, although for some histologic subtypes (such as liposarcoma) achieving microscopically-negative margins may be difficult and/or not feasible due to anatomic and technical constraints. Multimodal therapy with systemic or radiation therapies can be considered for appropriate histologic STS subtypes and clinical scenarios [5,6]. In general, patients with recurrent or metastatic retroperitoneal STS have inferior survival outcomes and prognosis compared to those with other sites of STS [3]. The 5- and 10-year disease-specific survival (DSS) rates for primary retroperitoneal sarcomas (RPS) are 50% and 35%, respectively. The rate of distant metastases is similar between RPS and extremity/trunk STS tumors and inferior prognosis for RPS is primarily due to higher local recurrence rates (LRR) [4,7]. The difference in prognosis, however, is not solely due to location but also the predominant histologic subtypes of RPS.

Beyond the technical challenges of surgery for RPS, peri-operative treatments are more challenging and have shown less benefit in RPS compared to other sites of disease. For instance, while radiation therapy (RT) provides local control benefit for patients with extremity sarcoma [8,9], the EORTC-62092 STRASS trial did not demonstrate a benefit of neoadjuvant RT for abdominal recurrence-free survival. Based on the literature, it is unclear if there is a survival benefit with cytotoxic chemotherapy for RPS. Common subtypes of RPS, particularly WDLPS, are considered chemotherapy resistant [10]. Additionally, the use of chemotherapy in patients with RPS is often limited by major multivisceral surgical resections, which often include prior history of nephrectomy, that may decrease dose and cumulative tolerability of systemic therapies [4]. Such circumstances highlight the need for more effective systemic therapies in the treatment of this family of tumors.

The addition of immunotherapy to the cancer treatment armamentarium has dramatically changed the landscape of cancer management in recent years. Specifically, immune checkpoint inhibitors (ICI) including anti-CTLA4 (ipilimumab, tremelimumab), anti-PD-1 (pembrolizumab, nivolumab), anti-PDL1 (atezolizumab, durvalumab), and anti-LAG3 (relatlimab) have led to durable treatment responses and improved survival for some patients with solid tumors such as melanoma, non-small cell lung cancer, renal cell carcinoma, bladder cancer, Hodgkin’s lymphoma, head and neck squamous cell carcinomas, and in mismatch repair-deficient cancers, amongst many others [11,12]. However, response rates to ICI in STS have been more modest, in part due to trials enrolling patients across multiple STS subtypes [13,14,15]. Thus, there has been a growing effort to identify predictors and biomarkers of response and resistance in patients with STS treated with immunotherapies and to elucidate the biology and mechanisms of response and resistance in order to improve oncologic outcomes [16,17,18,19]. The heterogeneity of STS carries significant implications, as sarcoma subtypes vary in mutational burden, immune infiltrate, and receptor status, all of which directly affect response to immunotherapy [20,21,22,23,24].

A pooled analysis of all clinical trials examining the effects of PD1 or PD-L1 antagonists in metastatic STS was performed in 2020 [25]. This review included 384 patients across nine clinical trials. One hundred and fifty-three patients (39.8%) received a PD1/PD-L1 antagonist as a single agent, with an overall response rate (ORR) and non-progression rate (NPR) of 15.1% and 58.5% respectively [18]. Focusing on STS subtypes that occur in the retroperitoneum, 61 DDLPS patients and 82 LMS patients were included. Amongst DDLPS patients, an ORR of 7.3% (CI 1.2–33.7) and NPR of 54.5% (CI 54.1–93.5) were noted. A similar response was found for LMS patients, with an ORR of 6.9% (CI 2.0–21.3) and NPR of 54.1 (CI 29.3–77.0) [25].

In this review, we aim to discuss the current understanding of immunotherapy as it applies to RPS, with respect to individual histologic subtypes and immune phenotypes. We will discuss the working knowledge of what may be contributing to responses (or lack thereof) seen in clinical trials of immunotherapy for RPS. For the purposes of this review, the most common histologies of the retroperitoneum (DDLPS, WDLPS, LMS, and SFT) will be discussed.

## 2. Predictors of Response to Immunotherapy in RPS

Recent studies to define characteristics of sarcomas which predict response or resistance to immunotherapy have included those examining tumor mutational burden, the tumor microenvironment (TME), and tumoral expression of immune checkpoint proteins PD-1, PD-L1, BTLA-4, CTLA4, and LAG3. With respect to the sarcoma TME, immune-related gene expression signatures (such as sarcoma immune class), intratumoral immune cells (such as tumor infiltrating lymphocytes, B-cells, natural killer [NK] cells, macrophages) and presence of tertiary lymphoid structures have been studied [19,21,22,26,27,28,29,30,31,32]. These studies have, in general, examined the STS family of neoplasms as a whole and the generalizability of the data from these studies with respect to RPS must be extrapolated from studies that included the most common RPS histologic subtypes.

### 2.1. Sarcoma Genetics and Tumor Mutational Burden (TMB)

**Figure 2:**
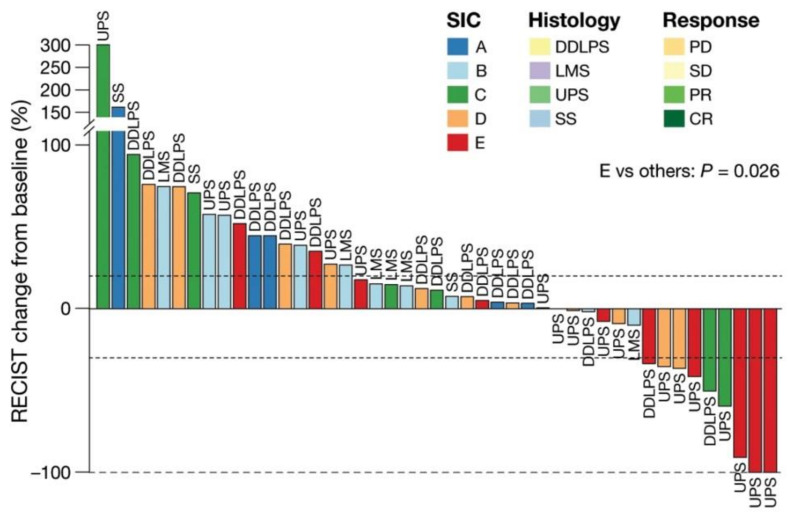
Distribution of SICs across sarcoma subtypes demonstrates histology-agnostic response to immunotherapy. Waterfall plot of patients from the SARC028 cohort demonstrating best response to pembrolizumab based on change of target diameter from baseline, as previously published by Petitprez et al. and the senior authors from this manuscript [21]. Colors represent the sarcoma immune class (SIC) of each tumor. Note SIC E class predominates response to pembrolizumab.

Greater response to immunotherapy is seen in tumors with high TMB (a high number of mutated genes) or that are mismatch repair (MMR)-deficient (which results in high TMB) [33,34]. Although the genetics of sarcomas are complex and vary significantly across histologies, TMB tends to be low [15,24,35,36] (Figure 2) while copy-number alterations and genomic rearrangements are common. While a handful of genes (TP53, Rb1, MDM2, ATRX) are found to be mutated across STS subtypes [15,35,37], average TMB is 1.1–2.5 mut/Mb, with only 5% of STS having TMB over 20 mut/Mb [4,15]. Additionally, MMR deficiency is uncommon among sarcomas [4,12,36,38]. In a large study of 304 sarcoma samples, only seven (2.3%) were found to be MMR-deficient (four tumors classified as sarcoma NOS and a single case each of pleomorphic rhabdomyosarcoma, epithelioid leiomyosarcoma, and malignant PEComa). Furthermore, MMR-deficient sarcomas had lower TMB compared to MMR-deficient carcinomas (median 28 versus 16, *p* = 0.006) [12].

Nacev et al. performed a comparative genetic analysis of over 2000 sarcoma samples across 22 histologic subtypes. This study corroborated that TMB is low but heterogenous between and within subtypes. None of the histologies commonly found in the retroperitoneum were classified as high TMB, and WDLPS had the lowest TMB of all subtypes [37]. The authors also performed unsupervised clustering and found that samples grouped by genomic mutations which were histotype agnostic, that is, distinct from histologic identity [37]. The exception to this were DDLPS and WDLPS, which were the dominant tumors with MDM2-CDK4 mutations and clustered as such. Conversely, LMS samples fell into the TP53, TP53+ATRX, TP53+RB1, TP53+ATRX+RB1, TERT, SMARCB1, CDKN2A/B, and NF1 genetic mutation clusters, suggesting a varied oncogenic pattern for this histologic subtype [37].

### 2.2. Tumor Infiltrating Lymphocytes (TILs)

Another factor studied in the landscape of RPS tumors have been TILs. Tseng et al. studied TILs specifically in retroperitoneal WDLPS and DDLPS [28] and found these to be comprised primarily of CD3+ T cells. Flow cytometry demonstrated that the majority of these were CD4+ T cells with a CD4+ to CD8+ ratio of 4.2:1 [28]. CD8+ cytotoxic T cells represented 20% of TILs based on flow cytometry and were present in all tumors with 65% of these cells expressing PD-1 [28]. Abeshouse et al. performed an analysis of The Cancer Genome Atlas (TCGA) examining the immune infiltrate of 206 sarcomatous tumors across six subtypes: DDLPS, LMS, undifferentiated pleomorphic sarcoma (UPS), myxofibrosarcoma, MPNST and synovial sarcoma [35]. Authors assigned each sarcoma type either a high or low immune infiltration score based on gene expression signatures. DDLPS had the highest macrophage and CD8+ T cells scores, whereas LMS had the highest PD-L1 score. DSS was then compared between the top versus bottom third of immune infiltrate scores across all subtypes. Across all tumors, NK cells were the only immune cell type to correlate significantly with DSS [35]. In DDLPS, elevated Th2 signature was associated with worse DSS [35]. In another study, TLS in non-responding patients was significantly more enriched with regulatory T cells (T regs) [39].

### 2.3. Sarcoma Immune Class, Intratumoral B-Cells, and Tertiary Lymphoid Structures

The TME, defined as the populations of cells surrounding tumor cells, include stromal, endothelial, adipocytes, cancer-associated fibroblasts, and immune cells as well as scaffold, collagen and cytokine signaling [22]. The study of the interactions between the primary tumor cells and TME has provided insights into the mechanisms related to overall tumor immunity and response to immunotherapy. Bagaev et al. performed a TCGA transcriptomic analysis (RNA sequencing) of over 10,000 cancer patients across 20 tumor types [26]. The authors defined four distinct TME subtypes based on transcriptomic signature: immune-enriched (fibrotic), immune-enriched (non-fibrotic), fibrotic, and depleted. The “immune-enriched” tumors demonstrated high expression of genes associated with pro- and anti-tumor immune infiltrates and angiogenesis. Conversely, the fibrotic and depleted subtypes showed lower expression of such genes. Importantly, the four subtypes correlated with patient response to immunotherapy at the pan-cancer level, regardless of anatomic origin of the tumors, with immune-enriched (non-fibrotic) tumors demonstrating superior responses to immunotherapy [26].

**Figure 3:**
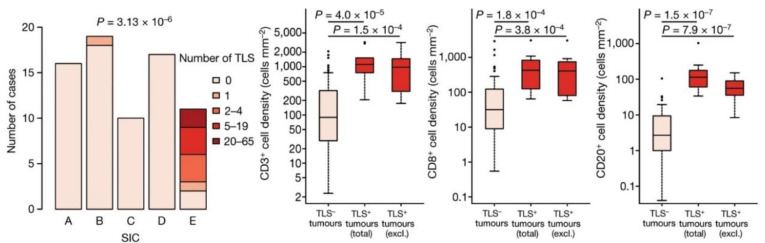
Sarcoma Immune Class E contains highest TLS density which correlates with response to immunotherapy. On left, number of tertiary lymphoid structures by SIC from 73 tumors from the National Taiwan University Hospital with *p*-value calculated by chi-squared test, as previously published by Petitprez, et al. and the senior authors from this manuscript [21]. Box and whisker plots characterize tumoral immune infiltrate according to TLS presence (TLS− n = 82, TLS+ n = 11). CD3+ densities are on left, CD8+ densities in center and CD20+ are on right. Note SIC E contains highest density of TLSs. (Large bar in box plot represents median, box represents interquartile range (IQR). Upper whisker extends to either minimal, maximum or third quartile plus 1.5 × IQR. Lower whisker extends to either maximal, minimum or first quartile minus 1.5 × IQR. *p*-value calculated by two-sided Mann–Whitney tests.).

It is important to note, however, that the TCGA study did not include sarcomas. An earlier study by Petitprez et al. studied the TME of 608 sarcomas, including DDLPS, LMS and UPS subtypes. This study established five distinct, and, importantly, histologic-agnostic, sarcoma immune classes (SIC) with regards to TME composition: immune-low (A and B), highly vascularized (C) and immune-high (D and E) (Figure 3) [21]. The authors showed that SIC E was associated with response to pembrolizumab and improved survival in the SARC028 trial [40]. Patients with SIC E tumors exhibited the highest response rate (50%, 5 of 10), followed by SIC D (25%, 3 of 12) and SIC C (22%, 2 of 9). No patients with SIC A (0 of 5) or SIB B (0 of 11) tumors responded to ICI [21]. SIC E and D tumors were also associated with improved OS (*p* = 0.029 and 0.011, respectively) [21]. Patients with SIC E tumors demonstrated improved progression-free survival (PFS) compared with patients with SIC A or B tumors (*p* = 0.023 and *p* = 0.0069, respectively) [21].

SIC E tumors were characterized by expression of B-cell lineage-associated genes and presence of B cells within the TME was associated with improved OS (log-rank test *p* = 4.25 × 10^−4^) [21]. Using an independent validation cohort, the authors then showed that SIC E tumors (but not SIC A, C, or D tumors nor most SIC B tumors) are characterized by the presence of tertiary lymphoid structures (TLS) [41,42]. TLS are ectopic lymphoid aggregates characterized by an inner zone of CD20^+^ B cells surrounded by CD3^+^ T cells, contain dendritic cells, and which have been shown to be associated with response to immunotherapy and better prognosis across cancer types.

Based on the findings of this study, the role of TLS in response to immunotherapy in sarcomas has become a topic of interest. The relationship between TME, B cells and TLS have been previously demonstrated in studies focused on other tumor types, such as melanoma [27]. The association between TLS and response to ICI across different tumor types is significant, as melanoma is classically considered to be an “immunologically hot” tumor with a known response to immunotherapy, whereas sarcoma has long been considered “immunologically cold.” The reproducibility of predictors of response between the two neoplasms suggests that TLS are important histology-agnostic predictors of response to ICI (Figure 3). In keeping with this concept, each STS subtype treated in SARC028 included tumors from each SIC, meaning the presence of TLS varies within any given STS subtype [22]. Thus, a solely histology-specific approach to selecting patients for ICI is limited as it would not account for variance in microenvironment and immune profile.

This concept was demonstrated in the PEMBROSARC trial (NCT02406781) and subsequent amendment which selected STS patients for ICI based on TLS+ status [39,43]. The initial Phase 2 trial assessed the combination of pembrolizumab with low-dose cyclophosphamide for 50 patients. Patients were divided into 4 cohorts: unresectable LMS, UPS, other sarcoma (others) and gastrointestinal stromal tumors. In total, 3 patients assessable for efficacy experienced tumor shrinkage in this histotype-dependent trial model [43].

The PEMBROSARC trial was then amended to include a fifth cohort whose tumors were TLS+ [39]. Response to treatment was significantly higher in this cohort. Initial 6-month NPR for 30 patients with TLS+ tumors was 40% (95% CI, 22.7–59.4) and objective response rate (ORR) was 30% (95% CI, 14.7–49.4). In contrast, 6-month NPR and ORR were 4.9% (95% CI, 0.6–16.5) and 2.4% (95% CI, 0.1–12.9), respectively, in the all-comer cohorts. The authors then performed exploratory analysis using spatial transcriptomics and multiplexed immunohistofluorescence. The tumors from responders were characterized by dense plasma cell infiltrates in tumor stroma [39]. These data suggest that baseline tumor characterization for intratumoral TLS may be an effective approach to guide patient selection for immunotherapy [39]. Importantly, histologic subtypes included in this study were WD/DDLPS, UPS and LMS, allowing for extrapolation of this data to patients with RPS.

### 2.4. Expression of Targetable Immune Checkpoints by Tumor Cells and TILs

The clinical relevance of PD-1 and PD-L1 on TILs and tumor cells has been historically controversial in sarcomas, initially due to their high heterogeneity across tumor subtypes. This is complicated by variance in the methodology of evaluation across studies and focus on expression of these markers by TILs versus tumor cells. At this time, there is no universal standard method to test for PD-1 pr PD-L1 expression. Differing methods to address this include sequencing methods (specifically transcriptomics), immunohistochemical evaluation, and antibody clone used for immunohistochemistry assessment) [31]. Results, therefore, must be interpreted in the context of the methods used and cell populations included. This section will discuss the presence of immune checkpoints on both tumor cells and TILs and methods used for assessment.

In a study of 225 STS samples, PD-L1 expression was noted on all tumors using immunohistochemistry, however with a broad range of positivity from 3% to 50% [32]. In DDLPS, PD-L1 expression was seen in 12% of tumors. In LMS, PD-L1 expression was observed in 13%. PD-L1 expression was associated with more PD-1+ TILs (*p* <  0.001) and higher tumor grade (*p* = 0.016). Percentage of PD-1+ TILs was also associated with worse 5-year OS in the absence of immunotherapy (*p* = 0.028) [32]. Given the rates of PD-L1 positivity of the tumors samples, the association of PD-L1 expression with PD-1+ TILs, and the associated worse OS, this lead the authors to suggest a rationale for checkpoint inhibition in patients with PD-L1-positive STS [32]. Additionally, STS has been shown to respond to ICI despite non-detection of PD-1 or PD-L1 on tumor cells [44]. Correlative analysis of the SARC028 trial tumors demonstrated higher densities of activated T cells (CD8+ CD3+ PD-1+), cytotoxic T cells, regulatory T cells and increased percentage of tumor-associated macrophages (TAM) expressing PD-L1 pre-treatment in responders to ICI compared with non-responders [44].

A broad comprehensive study utilizing immunohistochemistry of 1072 sarcoma specimens by Dancosk et al. noted genomically-complex sarcoma types had much higher numbers of TILs than translocation-associated sarcomas [30,31]. This lymphocytic infiltration was associated with better overall survival among the genetically-complex sarcomas. While PD-1 expression was again associated with worse survival in the absence of immunotherapy, the authors noted PD-1 expression was often associated with co-expression of either LAG-3 and TIM-3 (other immune checkpoints). The combination of LAG-3 and PD-1 inhibition has been found to confer greater PFS in metastatic melanoma compared to PD-1 inhibition alone [45]. Thus, this observation in sarcoma suggests a rationale for combination therapy targeting LAG-3 and/or TIM-3 in conjunction with PD-1 inhibition [29,30].

In another study of banked STS samples, TILs, including CD3+ T and CD56+ NK cells, were analyzed using flow cytometry and RNA sequencing, and findings corroborated using TCGA sequencing data [29]. Intra-tumoral T and NK cells demonstrated increased expression of activation and exhaustion markers when compared to samples from matched peripheral blood. In ex vivo studies, a combination of IL-15 (an activation cytokine) and TIGIT (a T and NK cell exhaustion marker) blockade increased cytotoxicity of NK and T cells against tumor cells. This study, like the Dancosk study, suggests targeting TILs with a combination of treatment to manipulate both activation and exhaustion may be an effective strategy to improve outcomes with regards to immunotherapy in sarcoma [29].

### 2.5. Histologic Subtypes of RPS and Relationship to Immunotherapy

Although available data suggests that histologic subtype is less predictive of response to ICI in patients with sarcoma compared to TME features such as SIC or presence of TLS, the most common subtypes of RPS have important characteristics and differences that bear mention. This section will focus on available data specific to the most common subtypes of RPS.

### 2.6. DDLPS (32–43% of RPS) and WDLPS (23–28% of RPS)

WDLPS are low-grade mesenchymal tumors comprised of mature adipocytes and stromal cells with at least focal cytologic atypia while DDLPS is composed of moderate to high-grade, non-lipogenic, undifferentiated tumor cells, the most aggressive variant of which is termed pleomorphic liposarcoma [46]. While WDLPS and DDLPS often coexist in patients with both primary and recurrent liposarcoma, each subtype can also be found in some patients without the other.

WDLPS and DDLPS contain giant chromosomes on their karyotype. The giant chromosomes contain genetic sequences of chromosome 12 and are characterized genetically by recurrent 12q13–q15 amplification. MDM2, located in 12q13, is thus amplified with induced high expression in almost all cases and act as the major oncogenic factor in WDLPS and DDLPS [46]. MDM2 is an E3-ubiquitin ligase that targets p53 for degradation by the proteosome and is a difficult target from a molecular perspective. Nutlin-3, an MDM2 antagonist, has a narrow therapeutic window with high toxicity [47]. Thus, an alternative to targeted molecular therapy, such as immunotherapy approaches, may be better tolerated by patients.

Cell division protein kinase 4 (CDK4) is a gene involved in cell cycle regulation located on Chr 12 and also amplified in 85% of WDLPS/DDLPS cases [48]. CDK4 inhibitors targeted against WDLPS and DDLPS, including palbociclib and abemaciclib, have been tested in Phase 2 studies with modest increases in PFS [49,50,51]. In the real-world setting, use of palbociclib in the perioperative setting was well-tolerated but no radiographic treatment responses were noted. Based on RECIST1.1, 47% of patients had stable disease (SD) as best response [52]. However, while anti-CDK4 monotherapy is associated with limited response, preclinical models have suggested overexpression of CDK4 may lead to induced expression of PD-L1 by tumor cells, paving the way to employ possible combination targeted therapy regimens [53].

DNA sequencing studies have demonstrated genetic subtypes within the histologic subtypes. For example, there are two genetic subtypes of DDLPS, termed “DDLPS chromosome instable” and “DDLPS quiet.” While both contain the classic MDM2 amplification, “DDLPS chromosome instable” has amplicons in chromosome 12q and also various other somatic copy number alterations (SCNAs) across the genome, to include CDK4, FRS2, HMGA2, JUN, CDKN2, ATRx, and NF1 [35,46]. Conversely the “DDLPS quiet” subtype does not contains these copy number variations [35]. This is important with regards to immunotherapy given the evidence that ICIs are more effective with high TMB. Thus, complexity of a tumor’s genome may contribute to response to immunotherapy for RPS.

In terms of clinically-applicable studies in checkpoint inhibition, the SARC028 trial (NCT02301039) was the first trial to assess for response to ICI in patients with sarcoma [40]. It was a multicenter, two-cohort, open-label, Phase 2 trial of pembrolizumab monotherapy in 40 patients with STS and 40 patients with bone sarcoma. STS subtypes included LMS, UPS, DDLPS, and synovial sarcoma [40]. The primary endpoint of overall response was not met, but 40% of patients with UPS, 20% of patients with DDLPS and 0% of patients with LMS had a response [40]. In the final analysis of expansion cohorts, DDLPS showed an ORR of 10% (4/39 patients) [54]. The subsequent correlative analysis found that patients with higher densities of activated T cells with PD-1 positivity and increased TAMs were more likely to have a response [44].

### 2.7. Leiomyosarcoma (18–23% of RPS)

Histologically, LMSs are sarcomas of smooth muscle origin with spindle cells with various levels of positivity for SMA, desmin and cytokeratin [55]. Retroperitoneal non-uterine LMS develops from the walls of retroperitoneal veins, most frequently the inferior vena cava [46]. LMS is considered a sarcoma with complex genetics, frequent tetraploidization, multiple SCNAs, whole-genome duplication, alternative telomere lengthening and frequent inactivation of tumor suppressor genes and genes responsible for DNA damage response. LMS is characterized by a complex genetic profile with TP53, RB1 and ATRX loss or mutation, as well as PTEN and DMD deletions [35,46,56].

Within the LMS histotype, there are three distinct genotypes: a uterine and two soft-tissue LMS (ST-LMS) subtypes [35,57,58]. Of the two ST-LMS subtypes, dedifferentiated soft-tissue genotype is characterized by significantly higher genomic instability due to loss of genes involved in homologous recombination and significantly worse OS. However, given the high TMB seen in the dedifferentiated subtype, it is observed to have relatively high TMB (relative to other sarcomas) and greater tumor immune infiltration [56]. The high TMB has been suggested as a target for immuno- or PARP-inhibitor therapy given the loss of genes involved with homologous recombination [56].

Bi-allelic loss of *PTEN* in LMS is associated with resistance to ICI, notably by decreasing the transcriptomic expression of immune genes such as *STAT3*, *PDCD1*, *CD8A*, *IFNG*, and *GZMA* [59,60]. Additionally, loss of *PTEN* in melanoma increases the expression of immunosuppressive cytokines, resulting in decreased T cell infiltration in tumors [61]. PTEN is a tumor suppressor gene that inhibits PI3K activity and downstream AKT/mTOR signaling, and thus, targeting this pathway in combination with ICI may be an interesting path forward. LMS also contains overexpression of certain microRNAs whose significance remains unknown [62].

LMS differs from DDLPS with regards to characterization of immune infiltrates, which have shown CD163+ M2 macrophages to be the most predominant cell type. M2 macrophages have also been shown to correlate with tumor grade [63]. In vitro, LMS cells have been found to produce M-CSF, thereby increasing CD163 positivity and driving macrophage polarization to the M2 phenotype. Additionally, whereas B cells and TLS are found in a minority LMS tumors, CD8+ T cell infiltrates have been found in more than 50% of tumor samples [21,25].

In the Alliance A091401 trial of nivolumab with or without ipilimumab in the metastatic setting, a few LMS responses were observed. Eighty-five eligible patients entered the study and received nivolumab monotherapy (43 patients) or nivolumab plus ipilimumab (42 patients) with median PFS of 1.7 months in the nivolumab monotherapy and 4.1 months in the nivolumab and ipilimumab combination arm, respectively. Of the 29 LMS patients enrolled, partial response (PR) was observed in 1 patient in the nivolumab monotherapy arm and two patients in the combination arm [64].

RPS LMS are vascular by nature and very responsive to antiangiogenics and there is ample evidence of a close relationship between angiogenesis and immune microenvironment [65,66,67]. In fact, in the sarcoma field, in a Phase 2 trial of pembrolizumab and axitinib combination, two patients with soft-tissue LMS were included, one patient had a PR which lasted for more than a year and the other patient experienced disease control for 6 months [68]. In contrast, no patient with uterine LMS responded. Thus, the combination of antiangiogenics and ICI may be effective in soft-tissue LMS.

### 2.8. SFT (5% of RPS)

SFT is the fourth most common sarcoma histotype in the retroperitoneum, although it is extremely rare. SFT is characterized by spindle cells and genetically is known to have Chr 12 inversions and q13 duplications, as well as NAB2-STAT6 fusion [35]. In general, SFT has a low malignant potential, with a 5-year LRR of 7%, a distant recurrence rate of 20%, and a 5-year cumulative incidence of 19% for disease-specific death. Although chemo-resistant, SFT is widely regarded as radiosensitive and sensitive to anti-angiogenics, and may therefore benefit from these therapies in combination with ICI [3,69,70,71,72].

### 2.9. Future Directions

While we have previously discussed select trials which have shaped our current understanding of immunotherapy with regards to RPS, there are others that bear mention and will inform future clinical trials (Table 1).

The Alliance A091401 trial was a multicenter, open-label, non-comparative, randomized, Phase 2 study of nivolumab alone (43 patients) or in combination with ipilimumab (42 patients) in patients with locally advanced, unresectable, or metastatic sarcoma [64]. As this trial relates to RPS, 29 LMS and five liposarcoma (LPS) patients were included. Primary endpoint was a confirmed objective response, which was observed in two (5%) patients in the nivolumab monotherapy group and six (16%) of patients in the combination therapy group. Median PFS was 1.7 months (95% CI 1.4–4.3) with nivolumab alone and 4.1 months (2.6–4.7) with combination therapy [64].

A single-center, single-arm, Phase 2 trial studying VEGF receptor tyrosine-kinase inhibitor axitinib plus the anti-PD-1 immune checkpoint inhibitor pembrolizumab (NCT02636725) was performed [68]. The primary endpoint was PFS at 3 months. Thirty patients were enrolled, of whom two had non-uterine LMS and two had DDLPS. The two LMS patients demonstrated either a partial or minor response while the DDLPS patients experienced a >50% and >100% increase in tumor burden while on therapy [68].

A non-randomized Phase 1/2 clinical trial of 37 patients with advanced sarcoma examined the safety and efficacy of doxorubicin and pembrolizumab in anthracycline-naïve patients with sarcoma, to include eight non-uterine LMS patients and four DDLPS patients (NCT02888665) [73]. Two patients with DDLPS had durable partial responses while LMS demonstrated mixed response with either <50% gain or loss of tumor based on objective response rate [73]. Of note, authors noted TILs were present in 21% of evaluable tumors on correlative studies. TILs were associated with inferior PFS (log-rank *p*  =  0.03) [73].

A single-arm, open-label, Phase 1/2 study tested the anti–PD-L1 antibody avelumab with trabectedin specifically in advanced LMS and LPS, NCT03074318) [74]. There were 11 LPS patients (nine with DDLPS) and of the LMS patients, 18 were non-uterine. The primary endpoint was ORR by RECIST 1.1. Among LPS patients, there were no RECIST 1.1 responses and seven (64%) had durable SD. Among LMS patients, four (17%) had PR and nine (38%) had SD [74]. Median PFS of the patients in Phase 2 (n = 23) was 8.3 months (95% CI, 2.5-NA). No difference by histological subtype was noted (*p* = 0.578) [74].

NCT03307616 examines neoadjuvant nivolumab monotherapy or nivolumab and ipilimumab combination therapy specifically in DDLPS of the retroperitoneum and recently closed to accrual [75,76]. Seventeen DDLPS patients are included with 2 years of follow up data. At preliminary results, the median pathologic response in the DDLPS cohort was 22.5% (95% CI 85–99) and median change in tumor size (radiographic response) was +9% [76]. At 2 years of follow up, the median PFS was 18 months for DDLPS (IQR = 8-NR), with 11 patients experiencing relapse and one patient with progressive metastatic disease on treatment [75].

Several studies are ongoing and recruiting at the time of this manuscript. Of note, at this time, there are no cell-based immunotherapy trials targeting RPS or common RPS histologic subtypes. NCT04420975 will examine use of nivolumab plus a biologic agent BO-112, a synthetic nanoplexed dsRNA designed to activate TLR3, RIG1 and MDA5 in all sarcomas, including RPS subtypes. This trial is active but not yet recruiting. A trial of lenvatinib and pembrolizumab has opened for advanced STS, to include a cohort devoted to LMS (NCT04784247). NCT04668300 will examine oleclumab (anti-CD73) and durvalumab for STS, including metastatic, relapsed or refractory DDLPS. APG-115 is a novel MDM2 inhibitor and will be studied in combination with pembrolizumab for advanced solid tumors with MDM2 or p53 mutations, such as DDLPS (NCT03611868). The ImmunoSarc trial (NCT03277924) is a Phase 1/2 trial examining the efficacy of sunitinib plus nivolumab or epirubicin, ifosfamide and nivolumab in several histologic subtypes, including LMS and SFT, and is currently recruiting.

A French Phase 2 trial (TORNADO) plans to examine the effect of neoadjuvant chemotherapy with retifanlimab on histologic response for previously untreated resectable RPS of any histology (NCT04968106). Retifanlimab is a humanized PD-L1 inhibitor that has been studied in Phase 3 trials for endometrial cancer, non-small cell lung cancer, and gastric and esophageal cancer, with orphan drug status for Merkle cell carcinoma and anal carcinoma. There are no published studies on retifanlimab in soft tissue sarcoma [77].

Notably, the MULTISARC (NCT03784014) is designed to randomize patients with metastatic STS to undergo next generation sequencing (NGS) of their tumor at diagnosis. Patients will then enter the Phase 2 second-line targeted treatment to genetic alterations of their tumor. The trial plans to recruit 960 patients and its goals are twofold: to determine (1) if NGS of tumors on a large scale is feasible and timely, and (2) if an NGS-guided therapy improves outcomes [78].

Given the results of prior studies, innovative approaches to combination therapies with ICIs are needed. The question of improving immunotherapy efficacy by combining it with RT has been raised. Dancosk et al. noted prior exposure to RT was associated with increased immune infiltrates [30]. Previous studies by Keung et al. demonstrated increased densities of CD3+, CD4+ and CD8+ TILs in extremity UPS following RT [77]. Additionally, PD-L1 expression was 0% prior to RT and rose to expression on 21% of tumor cells following RT. Canter et al. have demonstrated increased cytotoxic activity against sarcoma cells of tumor-infiltrating NK cells and increased intra-tumoral homing of peripheral NK cells following RT (10–20 Gy) [78]. In a clinical trial for immunocompetent canines with metastatic sarcoma, researchers demonstrated favorable PFS with RT of the primary tumor coupled with autologous transfer of activated NK cells [78]. While the STRASS trial did not demonstrate a primary benefit of neoadjuvant RT in the treatment of RPS, the abscopal effects of RT in the context of potential TIL recruitment for targeted immunotherapy should be considered for study in RPS.

The French trial STEREOSARC (NCT03548428) seeks to examine the relationship between stereotactic body RT (SBRT) and immunotherapy for oligometastatic STS. This trial is recruiting and is designed as a prospective, multicentric, randomized Phase 2 trial to evaluate the efficacy of SBRT with atezolizumab (a monoclonal antibody against PD-L1) versus SBRT only [79]. The authors plan to perform randomization 2:1 stratifying for LMS vs. LPS vs. undifferentiated sarcomas. The primary endpoint will be PFS according to RECIST V.1.1 rate at 6 months [79]. Secondary endpoints include toxicity, OS, quality of life evaluations and the impact of biomarkers on response rate. This trial is currently recruiting.

## 3. Conclusions

Great scientific strides have been made to understand the heterogenous family of neoplasms that comprise STS. Analyses on histologic subtype, tumor microenvironment, sarcoma immune class, tumor infiltrating lymphocytes and genetic analysis for tumor mutational burden have all provided critical insight into the environment and characterization of these tumors, with targets for potential immunotherapy. Among STS, RPS outcomes are currently limited given a lack of effective therapies. Although outcomes for RPS patients with ICI alone have been somewhat disappointing, molecularly tailored combination treatments hold promise for improving response rates and oncologic outcomes. Further studies are needed to harness and manipulate the tumor microenvironment and immune composition of these tumors in order to successfully apply immunotherapy.

## Figures and Tables

**Table 1:** Completed and ongoing immunotherapy trials that included patients with retroperitoneal sarcoma.

Study	Design	N	Population	Intervention and Comparator	Outcomes	Status
Alliance A091401	Phase 2 RCT	85	Locally advanced, unresectable, or metastatic sarcoma	Nivolumab versus Nivolumab + Ipilumumab	Objective response	Complete
NCT02636725	Phase 2 Single arm	30	Histologically confirmed sarcoma	Axitinib + pembrolizumab	PFS at 3 months	Complete
NCT02888665	Phase 1/2 Single arm	37	Anthracycline-naïve patients with advanced sarcoma	Doxorubicin + pembrolizumab	Safety Response rate by RECIST1.1	Complete
NCT03074318	Phase 1/2 Single arm	33	Advanced leiomyosarcoma and liposarcoma	Avelumab + trabectedin	Safety Response rate by RECIST1.1	Complete
NCT03307616	Phase 2, randomized, non-comparative trial	24	Surgically resectable retroperitoneal DDLPS or extremity/truncal UPS	Nivolumab versus Nivolumab + Ipilumumab	Pathologic and radiographic response	Closed to accrual
NCT04420975	Phase 1	TBD	STS patients undergoing preoperative radiotherapy	Nivolumab + BO-112	Safety	Active
NCT04784247	Pilot	TBD	Metastatic STS	Lenvatinib + pembrolizumab	ORR	Recruiting
NCT04668300	Phase 2	TBD	Relapsed/refractory STS	Oleclumab + durvalumab	Response rate by RECIST1.1	Recruiting
NCT03611868	Phase 1/2	TBD	Advanced solid tumors with MDM2 or p53 mutation	APG-115 + pembrolizumab	Safety Response rate by RECIST1.1	Recruiting
TORNADO NCT04968106	Multicenter, prospective, open-labeled, 2-arm, non-comparative randomized phase II	TBD	Resectable STS	Doxorubicin + ifosfamide versus Doxorubicin + ifosfamide and retifanlimab	Pathologic response following surgical resection	Recruiting
MULTISARC NCT03784014	Phase 3 Randomized multicenter study	TBD	Advanced STS	Nilotinib Ceritinib Capmatinib Lapatinib Trametinib Trametinib + Dabrafenib Olaparib + Durvalumab Palbociclib Glasdegib or TAS-120 based on NGS results versus Standard treatment	Feasibility, 1 year PFS	Recruiting
STEREOSARC	Open label, Phase 2, prospective, multicentric, randomized study 2:1	TBD	Oligometastic STS	Atezolizumab + SBRT Vs. SBRT	6 month PFS	Recruiting

DDLPS, dedifferentiated liposarcoma; NGS, next-generation sequencing; PFS, progression-free survival; RCT, randomized controlled trial; SBRT, stereotactic body radiation therapy; STS, soft tissue sarcoma; TBD, to be determined; UPS, undifferentiated pleomorphic sarcoma.
